# Evaluating Surgical Safety and Quality Assurance by Conducting an Audit of WHO Checklist Implementation in a Tertiary Care Hospital

**DOI:** 10.7759/cureus.82212

**Published:** 2025-04-13

**Authors:** Amna Batool, Ahmed Naeem, Khaleeq Ur Rehman

**Affiliations:** 1 Surgery, Fatima Memorial Hospital (FMH) College of Medicine and Dentistry, Lahore, PAK; 2 Anesthesiology, Fatima Memorial Hospital (FMH) College of Medicine and Dentistry, Lahore, PAK; 3 Urology, Fatima Memorial Hospital (FMH) College of Medicine and Dentistry, Lahore, PAK

**Keywords:** healthcare, operation theater, surgery, surgical safety, surgical safety checklist

## Abstract

Background: Surgical care is an integral part of healthcare aimed at preventing surgical complications. To ensure safety during surgical procedures, the World Health Organization (WHO) introduced a surgical safety checklist (SSC). This checklist has been shown to significantly reduce the risk of complications and adverse outcomes caused by poor practices or negligence during surgeries.

Methodology: Following ethical approval, an audit was conducted on 200 major surgeries performed at Fatima Memorial Hospital in Lahore. The WHO SSC was converted into a “Yes/No” questionnaire using Google Forms. The audit was conducted over two months, from July to August 2024. Compliance with the three checklist phases (Sign In, Time Out, and Sign Out) was analyzed using the IBM SPSS Statistics for Windows, Version 26.0 (Released 2019; IBM Corp., Armonk, NY, United States).

Results: The Sign Out phase showed the highest compliance (178, 89%), followed by the Sign In phase (164, 82%). The lowest compliance was observed in the Time Out phase. Full compliance (100%) was recorded for obtaining patient consent, following anesthesia protocols, ensuring instrument sterilization, and team member introductions, indicating a safe and collaborative surgical environment.

Conclusion: Overall, checklist compliance was satisfactory but could be improved through targeted awareness initiatives. While the study did not assess surgical outcomes directly, it highlighted risks associated with incomplete adherence to the WHO checklist.

## Introduction

Surgical care is vital in every healthcare system, with an estimated 234 million procedures performed globally each year. However, surgical interventions are not without risk; complications occur in approximately 3%-16% of all surgeries [1,2]. This translates to one adverse outcome in every 300 procedures, with an associated mortality rate ranging from 0.4% to 0.8%. Notably, the incidence of surgical complications is significantly higher in developing countries than in developed nations [3].

In 2007, the World Health Organization (WHO) launched its global campaign “Safe Surgery Safe Lives,” identifying critical factors that significantly impact patient outcomes. These included inadequate anesthesia safety practices, preventable surgical infections, and poor communication among operating room personnel [4]. For these reasons, WHO developed the Surgical Safety Checklist (SSC) to promote standardized safety protocols within surgical theaters. In 2008, WHO published an article demonstrating that SSC implementation led to a significant reduction in surgical complications and adverse events. Complication rates dropped from 11% to 7%, and surgical mortality decreased by 53% [5]. Subsequent studies have shown that the SSC not only reduces perioperative complications but also enhances communication among operation theater teams [6]. A direct correlation has been observed between improved clinical outcomes and both the adoption and consistent use of the SSC [7].

The objective of this study was to evaluate the quality of SSC implementation in a tertiary care hospital in Lahore, to enhance surgical outcomes and reduce complications resulting from inadequate surgical practices.

## Materials and methods

A prospective observational study was conducted using a consecutive sampling technique at the operating theater of Fatima Memorial Hospital (FMH) in Lahore, from July to August 2024. A total of 200 randomly selected surgical procedures were audited. Inclusion criteria consisted of patients admitted to FMH who underwent major surgery. Minor surgeries and procedures under local anesthesia were excluded from the sample. The WHO SSC was adapted into a “Yes/No” questionnaire using Google Forms. Data were collected through direct observation and review of complete SSC charts, which were documented digitally using Google Forms as the audit proforma. The study received ethical approval from the IRB Committee of FMH College of Medicine and Dentistry, Lahore, registered under IRB-1430. The collected data were analyzed using IBM SPSS Statistics for Windows, Version 26.0 (Released 2019; IBM Corp., Armonk, NY, United States) and presented as percentages and bar charts.

Standards of audit

The WHO developed safety guidelines to strengthen the commitment of healthcare staff and address key safety concerns in surgical settings. The SSC is divided into three critical phases: before the induction of anesthesia, before the surgical incision, and before transferring the patient to the recovery room. Ideally, 100% compliance with the checklist is expected, in accordance with the standards outlined on the official WHO website [8]. The standard structure of the SSC is presented in Table 1.

**Table 1:** Standard structure of the surgical safety checklist drafted by the WHO

Sr. no.	Standards	Target	Evidence	Data source	Exception
Part I: Before the induction of anesthesia					
1	Confirm patient's identity, procedure, and consent	100%	WHO guideline	Direct observation/interview	None
2	Mark surgical site	100%	WHO guideline	Direct observation/interview	None
3	Check anesthesia machine and medications	100%	WHO guideline	Direct observation/interview	None
4	Known allergy	100%	WHO guideline	Direct observation/interview	None
5	Difficult airway/aspiration	100%	WHO guideline	Direct observation/interview	None
6	Risk of bleeding > 500 mL (7 mL/kg in children)	100%	WHO guideline	Direct observation/interview	Minor procedures with low risk of bleeding
Part II: Before the surgical incision					
7	All team members introduce themselves by name and role	100%	WHO guideline	Direct observation/interview	None
8	Surgeon, anesthetist, and registered practitioner confirm patient's name, planned procedure, site, and position	100%	WHO guideline	Direct observation/interview	None
9	Critical/unanticipated steps the surgeon may announce to the team	100%	WHO guideline	Direct observation/interview	None
10	Patient-specific concerns of anesthetist	100%	WHO guideline	Direct observation/interview	None
11	Nurse confirms sterility of instrumentation	100%	WHO guideline	Direct observation/interview	None
12	Antibiotic prophylaxis within the last 60 minutes	100%	WHO guideline	Direct observation/interview	Not applicable if no prophylaxis is indicated
13	Essential imaging displayed	100%	WHO guideline	Direct observation/interview	Not applicable if no imaging required
Part III: Before transferring the patient to the recovery room					
14	Nurse verbally confirms the name of the procedure	100%	WHO guideline	Direct observation/interview	None
15	Confirm instruments, swabs, and sharps counts are complete	100%	WHO guideline	Direct observation/interview	Emergency situations where counting is not feasible
16	Specimens labeled by patient's name	100%	WHO guideline	Direct observation/interview	No specimen collected
17	Address any equipment problems	100%	WHO guideline	Direct observation/interview	If all equipment is functional
18	Report key concerns for recovery room professionals	100%	WHO guideline	Direct observation/interview	None

## Results

During the audit, efforts were made to ensure checklist compliance aligned with the WHO standards. Similar to the checklist, the audit questionnaire was also divided into three components: Sign In, Time Out, and Sign Out. In the Sign In phase, strong adherence to certain checkpoints was observed. Confirmation of the patient’s identity, type of procedure, and consent was completed in all 200 cases (100%). Functional checks of the anesthesia machine, pulse oximeter, and necessary medications were also consistently performed. However, documentation of known allergies was present in only 128 cases (64%), leaving 72 patients (36%) without this vital information recorded prior to surgery. Low compliance was observed in surgical site marking, completed in only 84 cases (42%). Moreover, the risk of significant blood loss was documented in only 116 cases (58%), indicating the need for improved identification and documentation of high-risk possibilities.

**Figure 1:**
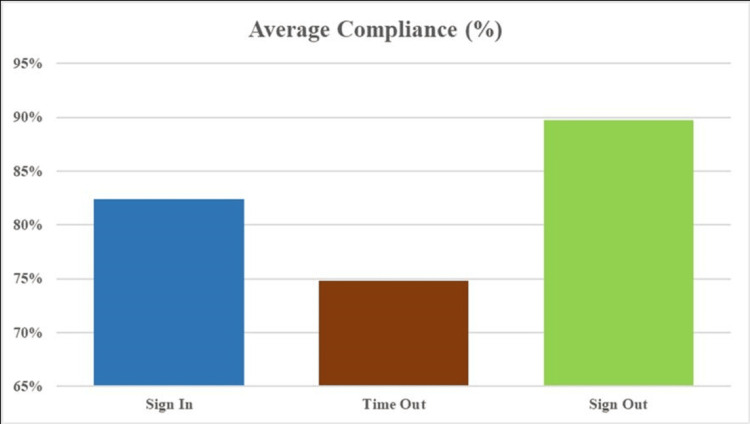
Average compliance of all three phases of the checklist

In the Time Out phase, team member introductions were completed in 186 cases (93%). Critical components, like anticipated blood loss, unanticipated procedural steps, and estimated procedure duration, were communicated with high compliance in approximately 198 cases (99%). Adherence to antibiotic administration for the prevention of postoperative infections was documented in only 142 cases (71%), leaving 58 cases (29%) at risk of developing infections after surgery. Compliance with the display of essential imaging was notably low, observed in only 95 cases (46.8%), indicating a need for improved clinical preparation. During the Sign Out phase, exemplary adherence was noted. Key steps such as verifying the name of the procedure, completing instrument, sponge, and needle counts, and addressing recovery room concerns were consistently followed. Specimen labeling showed moderate adherence (146 cases, 73%), while equipment issue documentation had higher compliance (171 cases, 85.5%), though with some room for improvement. Overall, the Sign Out phase demonstrated the highest compliance (179 cases, 89.70%). The Time Out phase showed the lowest adherence (149 cases, 74.80%), while the Sign In phase showed moderate adherence (165 cases, 82.40%), as illustrated in Figure 1. A summary of the overall audit results is presented in Table 2.

**Table 2:** Checklist for achieved and skipped items

Sr.	Standards	Achieved		%	Skipped	%
Part I: Sign In						
1.	Confirm patient's identity, procedure, and consent	200		100	0	0
2.	Mark surgical site	14		42.4	19	57.5
3.	Anesthesia machine and medication check	200		100	0	0
4.	Pulse oximeter on the patient and functioning	200		100	0	0
5.	Known allergy	128		64	72	36
6.	Difficult airway or aspiration risk	145		72.5	55	27.5
7.	Risk of > 500 mL blood loss (7 mL/kg in children)	119		59.5	81	40.5
Part II: Time Out						
8.	All team members introduce themselves by name and role	187		93.5	13	6.5
9.	Surgeon, anesthetist, and nurse confirm verbally patient's name, procedure, and site of incision	200		100	0	0
10.	Antibiotic prophylaxis within the last 60 minutes	141		70.8	58	29.1
11.	Critical/unanticipated steps	198	99		2	1
12.	How long will the case take	199	99.5		1	0.5
13.	Anticipated blood loss	147	73.5		53	26.5
14.	Patient-specific concern for anesthetist	199		99.5	1	0.5
15.	Nurse confirmation about the sterility of instrumentation	200		100	0	0
16.	Nurse confirmation about equipment issues or any concerns	156		78	44	22
17.	Essential imaging displayed	30		46.8	34	53
Part III: Sign Out						
18.	The name of the procedure	200	100		0	0
19.	Completion of instruments, sponge, and needle counts	200	100		0	0
20.	Specimen labeling (read specimen labels aloud, including patient's name)	146	73		54	27
21.	Whether there any equipment problems to be addressed	171	85.5		29	14.5
22.	Report key concerns for the recovery room professionals	200		100	0	0

## Discussion

The WHO SSC (as shown in Table 2) is structured into three critical time points, which align with key phases of the surgical process. These time points are designed to enhance team communication, prevent avoidable errors, and ultimately improve surgical outcomes. Despite their importance, ensuring consistent implementation remains a significant challenge.

In this study, the Sign In phase showed a significant compliance rate. Confirmation of patient’s data was fully adhered to, aligning with the global standards and findings from other studies [9,10]. Similarly, safety checks for anesthesia were also fully complied with. However, areas such as allergy documentation, aspiration risk assessment, and anticipated blood loss estimation showed lower compliance. These elements are essential for surgical safety, and their consistent evaluation is necessary.

The Time Out phase showed relatively lower compliance compared to the other two phases. Notably, the introduction of team members by role and name was performed more consistently in this study compared to similar studies conducted in other settings [11-13]. However, significant gaps were observed in antibiotic prophylaxis administration, as many patients received antibiotics outside the recommended timeframe, potentially contributing to a higher risk of postoperative infections. A critical oversight during this phase was the lack of action regarding the display of essential imaging, which is vital for ensuring surgical precision and patient safety. Failure to review imaging at this stage can negatively impact surgical decision-making [14-16]. 

The Sign Out phase showed the highest compliance among the three checklist phases. This phase plays a vital role in confirming surgical instrument counts and addressing any recovery-related concerns. During this phase, the nurse verbally confirms the name of the surgical procedure, ensures that all surgical instruments are accounted for, and verifies the specimen labels. Two of these checkpoints were fully adhered to, reflecting an established approach to surgical safety. However, specimen labeling showed only moderate compliance and requires further improvement. Accurate labeling is essential to confirm which patient underwent which procedure and to correlate the surgical specimen with the appropriate diagnosis. Several studies have identified similar gaps, often attributed to staff shortages or workload pressures in the operating room [17,18].

All the equipment-related concerns were efficiently identified and reported to the recovery room specialists. However, minor lapses were noted in the formal handover of concern reports, as indicated in Table 2. Despite this, the Sign Out phase demonstrated the highest overall compliance, highlighting the efficiency and vigilance of nursing staff in managing critical surgical transitions.

This study presents both strengths and limitations. This study presents both strengths and limitations. Among its strengths, all surgical departments involved were actively engaged and motivated to adhere to safety protocols. Strong teamwork and communication were observed among operating room personnel, and the paramedical staff fulfilled their roles diligently. Data collection was carried out by a trained research team and reviewed by an experienced supervisor, contributing to the reliability of the findings. Data collection was carried out by a trained research team and reviewed by an experienced supervisor. However, the study has some limitations. It was conducted in a single institutional setting, which limits the generalizability of the results. The short audit period and relatively small sample size also restrict the depth and scope of the conclusions drawn.

## Conclusions

Healthcare checklists have the potential to improve patient outcomes. While the SSC has been shown to enhance surgical safety, its effectiveness largely depends on consistent and thorough implementation in clinical settings. This study demonstrated moderate-to-high compliance with the SSC, indicating promising progress toward reducing surgical complications. However, the adherence levels still fall short of global standards, suggesting that risks remain. To bridge this gap, it is recommended that awareness programs be initiated to emphasize the importance of following the SSC. Recognition and awards should be given to surgical teams that consistently adhere to safety checklists. Ultimately, enhanced implementation of surgical safety protocols can lead to a reduction in preventable complications, contributing to lower morbidity rates and better overall outcomes for patients and society.
